# A Mass Transfer Analysis of Competitive Binding of Pb, Cd, and Zn from Binary Systems onto a Fixed Zeolite Bed

**DOI:** 10.3390/ijerph16030426

**Published:** 2019-02-01

**Authors:** Ivona Nuić, Marina Trgo, Nediljka Vukojević Medvidović, Marin Ugrina

**Affiliations:** Faculty of Chemistry and Technology, University of Split, Ruđera Boškovića 35, 21 000 Split, Croatia; mtrgo@ktf-split.hr (M.T.); nvukojev@ktf-split.hr (N.V.M.); mugrin@ktf-split.hr (M.U.)

**Keywords:** binary systems, lead, cadmium, zinc, competition, overshooting, overall mass transfer coefficient

## Abstract

The low-cost natural zeolite clinoptilolite was successfully applied for the competitive removal of Pb, Cd, and Zn from binary (Pb + Zn) and (Cd + Zn) aqueous solutions at different Pb/Zn and Cd/Zn concentration ratios. The obtained efficiency was in the range of 79.0–85.0%, and was similar for both systems, indicating that no loss in capacity was observed for six successive sorption-desorption cycles. In both systems, after the breakthrough, competition between the ions occurs, leading to overshooting in the Zn’s initial concentration, indicating displacement of already-bound Zn with Pb and Cd from the feeding solutions. The Zn exceeded its initial concentration up to 2.3 times in the presence of Pb, and up to 1.2 times in the presence of Cd. The film diffusion was pronounced as the slowest step responsible for the overall process rate. The overall mass transfer coefficient (*K*_a_) shows higher values for the (Cd + Zn) system compared to the (Pb + Zn) one due to reduced competition and ions migration. An SEM-EDS analysis confirmed a higher amount of bound Pb and Cd compared to Zn, and a mapping analysis revealed the equal distribution of all ions onto the zeolite surface.

## 1. Introduction

Rapid economic development and changes in lifestyle intensify emissions of various pollutants into the environment, especially those of heavy metals that have become a major environmental concern at the global scale [1,2,3,4]. Increased levels of different heavy metals have been found widely in various environmental media, since they are present in industrial, mining, municipal, urban, and rural runoff [5]. The main anthropogenic sources of heavy metals are industrial plants, such as battery manufacturing, explosives, photographic materials, plating, petroleum-refining, metal-processing, fertilizers and pesticides industry, textile, paint manufacture, pigments, and mining plants as well as agricultural activities, where pesticides and fertilizers containing heavy metals are widely used [6]. They can be washed away by runoff into surface waters, thereby resulting in considerable groundwater pollution causing a limited water supply [7,8,9]. Cadmium and lead as toxic metals are included in the list of priority substances in the field of water policy that has been established by the European Parliament in its Decision No. 2455/2001/EC [10]. These metals, together with zinc, are the most common heavy metals present in a variety of industrial wastewaters and their removal from industrial effluents is necessary due to their high toxicity and carcinogenicity [8,11,12,13]. To minimize their concentration below the maximum allowed one, tertiary treatment processes, such as adsorption, ion exchange, and membrane techniques, have been developed [14]. The high price of these techniques limits their application. The cost-effectiveness and technical applicability of these techniques has led researchers in the recent decades to search for the most suitable and easily available sorbents. Different low-cost sorbents, such as waste materials from the food and agricultural industry, have been applied in heavy metal removal [11,14,15,16,17,18,19]. Lakshmipathy et al. 2015 used watermelon rind in Pb removal and achieved efficiency in the range ≈16.0–72.5% under various experimental conditions [20]. Sivaprakash et al. 2010 successfully applied the marine alga *Sargassum Tenerrimum* in Cu removal with efficiencies in the range ≈51.0–62.5% [21]. Although acceptable removal efficiencies of heavy metals onto bio-sorbents have been obtained, a loss of bio-sorption capacity often occurs after several cycles [21,22]. The zeolites, low-cost silicate minerals widely distributed in nature, have been extensively studied in wastewater treatment for the removal of trace quantities of heavy metals [11,23,24,25]. Among the most studied natural zeolites, clinoptilolite is the most abundant one in the removal of heavy metals due to its largest deposits, high cation exchange capacity, and great selectivity [17,26,27,28,29]. The advantage of natural zeolites compared to waste and bio-sorbents is that they can be regenerated and thus consecutively reused without a noticeable loss in capacity, as well as safely disposed of after exhaustion by solidification into building materials [8,30,31,32]. Our earliest investigations have been focused on the removal of different heavy metals (Zn, Pb, Cu) onto zeolite by the batch method, as it provides an assessment of essential operating parameters in a short time [33,34]. Since the column performance allows for a treatment of larger amounts of wastewater and thus enables the industrial application, our further research was based on the column flow-through method from single solutions, where the obtained clinoptilolite removal efficiency for Pb was in the range ≈84.0–94.0% [35,36,37], and for Zn in the range ≈62.0–89.0%, for different experimental conditions [38]. A column filled with a natural zeolite fixed bed is one of the most effective designs for successive sorption/elution cycles, allowing for recovery and reuse of the sorbent and bound metals [35,36,37,38,39,40]. Since industrial wastewaters usually contain more than one heavy metal ion [17], their removal from multi-metal systems is required, which is very complex due to interferences and the competition phenomenon for the same sorption active sites. Accordingly, our recent investigations have focused on the removal of Pb and Zn from binary systems, where the obtained total removal efficiency was slightly lower due to ions competition [41,42,43]. Ion exchange from multi-metal solutions is challenging because different metals feature different affinities toward the sorbent as well as sorbent selectivity toward ions [24,39,41,42,43,44,45,46,47]. Mier et al. in 2001 found that Mexican clinoptilolite preferentially removes Pb over Cd in the column but not in the batch method. This implies that metal ions that diffuse faster to the available active sites may be removed in a greater extent than those with favourable equilibrium partitioning [26]. Moreover, when two components are present in the solution, in the initial stage they compete for the available sorption sites; however, over time, in the absence of free binding sites, the component with higher affinity displaces the one with lower affinity, causing its maximum effluent concentration to exceed (overshoot) the influent concentration [46,48]. Accordingly, in our previous investigations [41,43], overshooting in the (Pb + Zn) system has been determined for zinc ions. An overshooting in the Zn exit concentration in the presence of Cd was also observed on a *Sargassum algal* bio-sorbent by Figueira et al. 2000, but not on a *Sargassum fluitans* bio-sorbent by Naja et al. 2006 [45,47]. All these findings point to the unpredictability of metal interaction in multicomponent solutions and imply that there is no certain rule for explaining their behaviour without detailed research [39]. The intention of the present study has been to determine and clarify the interaction of metal ions in two binary systems, (Pb + Zn) and (Cd + Zn), with different affinities and physical and chemical properties (hydrated ionic radius, electronegativity, and energy of hydration) [29,49], depending on their initial concentrations. This paper additionally contributes to our understanding of the diffusion phenomenon and the mass transfer behaviour [50,51,52], which is important for obtaining more advanced and efficient column operations. For that purpose, the overall mass transfer coefficient (*K*_a_) in the fixed zeolite bed has been evaluated to provide useful descriptions of diffusion in complex multi-metal systems.

## 2. Materials and Methods

### 2.1. Zeolite Sample

The natural zeolite-rich rock sample originates from the Zlatokop deposit in Vranjska Banja (Serbia). The sample was milled and sieved to the particle size *d*_p_ = 0.6–0.8 mm, rinsed in ultrapure water in order to eliminate impurities possibly present, dried at 60 °C, and stored in a desiccator. According to the semi-quantitative mineralogical analysis (SEM, XRD), the zeolite contains up to 80% of clinoptilolite as the major mineralogical component, with quartz as an impurity. The theoretical exchange capacity of the zeolite sample has been calculated from the chemical composition and equals 1.411 mmol/g [41]. The characterization of the raw zeolite sample by the X-ray powder diffraction method (XRPD), a scanning electron microscopy and energy dispersive X-ray analysis (SEM-EDS), a thermal analysis (TG-DTG), and Fourier-transform infrared spectroscopy (FTIR) has been performed and published in our previous paper [53].

### 2.2. Solutions

The heavy metal binary solutions (Pb + Zn) and (Cd + Zn) were prepared in ultrapure water by dissolving appropriate amounts of Pb(NO_3_)_2_, Cd(NO_3_)_2_·4H_2_O, and Zn(NO_3_)_2_·6H_2_O in ultrapure water, without pH adjustment. The total concentration of the binary solutions was constant and equaled *c*_o_ ≈ 1 mmol/L, but with different *c*_o_(Pb)/*c*_o_(Zn) and *c*_o_(Cd)/*c*_o_(Zn) concentration ratios (Pb/Zn and Cd/Zn) in the range 0.14–2.15. The regeneration solution of *c*(NaNO_3_) = 176.5 mmol/L has been prepared by dissolving NaNO_3_ salt in ultrapure water.

### 2.3. Column Studies

**Figure 1:**
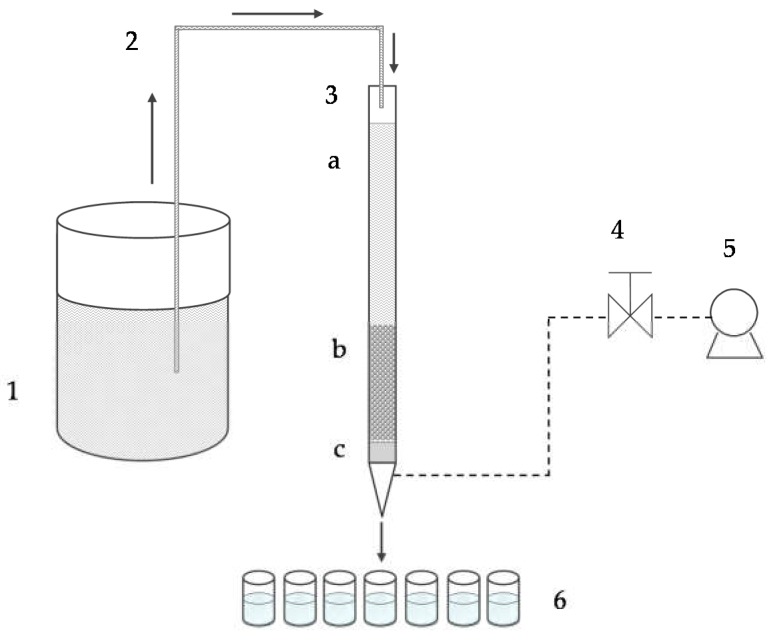
A schematic representation of the laboratory column experiment: (1) binary feeding solution, (2) glass tube, (3) glass column, (**a**) layer of the feeding solution, (b) zeolite fixed bed of *H* = 8 cm, (**c**) glass wool for supporting the zeolite packed bed, (4) flow rate setting, (5) vacuum pump, and (6) effluent samples.

Laboratory column tests (Figure 1) were performed isothermally at the ambient temperature (23 ± 2 ºC) in a 50-cm-long glass column of 1.2 cm internal diameter, filled with the zeolite sample up to a bed depth of *H* = 8 cm, which yields the mass *m* = 5.9 g. The zeolite bulk density *ρ* and fixed bed porosity *ɛ* were 0.699 g/cm^3^ and 0.693, respectively.

Binary feeding solutions were fed through the bed in the down-flow mode at the constant flow rate *Q* = 1 mL/min using the vacuum pump. The bed depth, temperature, pressure, and flow rate were kept constant to avoid contraction or swelling of the material in the column. All cycles were performed on the same zeolite layer, since the column’s performance enables its reuse through the recovery of heavy metals by regeneration. In all experiments, the effluent samples were periodically collected and analyzed for Pb, Cd, and Zn concentrations (AAS, IC) and pH values (a Mettler Toledo pH meter).

### 2.4. Scanning Electron Microscopy and Energy Dispersive Spectroscopy (SEM-EDS) Characterization of Saturated Zeolite

The surface structure and elemental composition of the natural zeolite saturated with lead, cadmium, and zinc was observed by scanning electron microscopy (SEM) and energy dispersive X-ray (EDS) analysis on a JEOL JSM-6610LV microscope (JEOL Ltd., Tokyo, Japan) in Belgrade (Serbia). A few grains of saturated zeolite were taken from the top of the zeolite layer in the column each time before the regeneration cycle. Since the samples are non-conductive, they were coated with a thin layer of gold and subjected to SEM observations at magnifications from 100 to 5000. SEM revealed information about the surface morphology, while EDS provided a spot analysis of the elemental composition at different features that were observed in the SEM micrographs.

## 3. Results and Discussion

### 3.1. The Comparison of Breakthrough Curves for (Pb + Zn) and (Cd + Zn) Binary Systems

**Figure 2:**
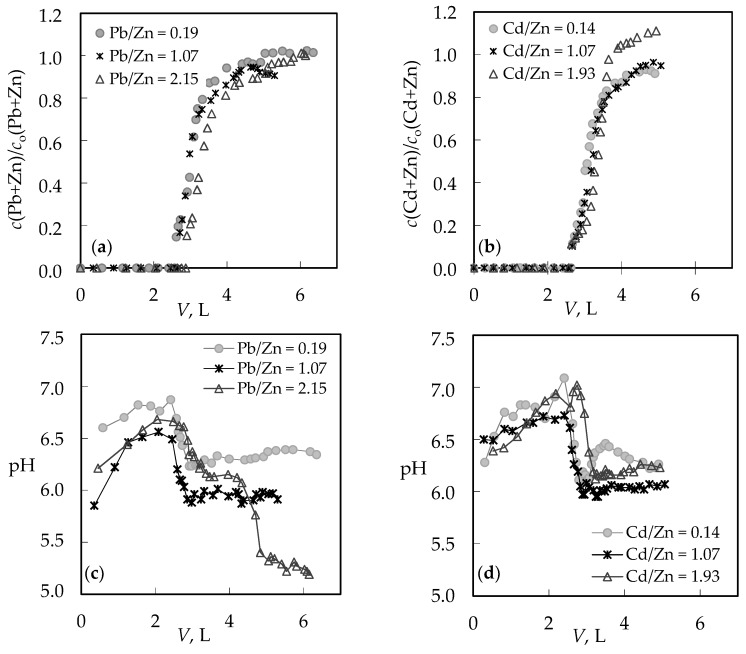
Breakthrough curves for different Pb/Zn and Cd/Zn concentration ratios expressed as: (**a**,**b**) the effluent and influent concentration ratios versus volume of the treated solution; (**c**,**d**) pH changes during service cycles versus volume of the treated solution.

The comparison of breakthrough curves for the (Pb + Zn) and (Cd + Zn) binary systems is given in Figure 2a,b by plotting effluent (*c*) and influent (*c*_o_) concentration ratios versus volume (*V*) of the treated solution. The typical S-shape breakthrough curves for both binary systems are evident, indicating successful removal of Pb, Cd, and Zn onto zeolite, and properly chosen experimental conditions.

**Figure 3:**
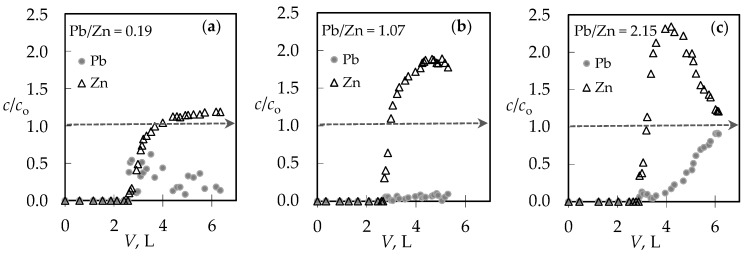
The breakthrough curves for each metal ion in the binary (Pb + Zn) solution for different Pb/Zn ratios: (**a**) Pb/Zn = 0.19; (**b**) Pb/Zn = 1.07 [43]; and (**c**) Pb/Zn = 2.15. Note: *c*/*c*_o_ = *c*(Pb)/*c*_o_(Pb) or *c*(Zn)/*c*_o_(Zn).

**Figure 4:**
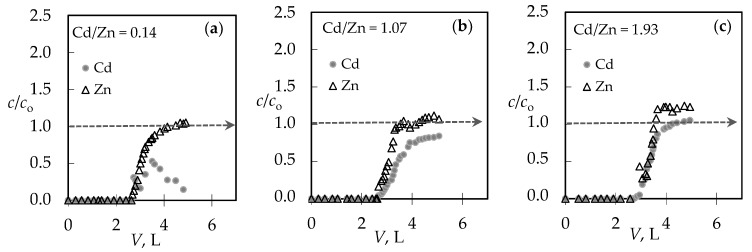
The breakthrough curves for each metal ion in the binary (Cd + Zn) solution for different Cd/Zn ratios: (**a**) Cd/Zn = 0.14; (**b**) Cd/Zn = 1.07; and (**c**) Cd/Zn = 1.93. Note: *c*/*c*_o_ = *c*(Cd)/*c*_o_(Cd) or *c*(Zn)/*c*_o_(Zn).

For the (Pb + Zn) binary system, the total breakthrough curves almost overlap, while for the (Cd + Zn) binary system for Cd/Zn = 1.93 overshooting in the total concentration has been observed where *c*(Cd + Zn)/*c*_o_(Cd + Zn) is higher than 1.0. Breakthrough (*t*_B_, *V*_B_) and exhaustion (*t*_E_, *V*_E_) points are slightly delayed for the highest Pb/Zn ratio. In the (Cd + Zn) system, the difference in achieving the breakthrough point, depending on Cd/Zn ratios, is negligible, while the exhaustion point is slightly delayed for the equimolar solution (Table 1). The monitoring of pH values in the effluents can be very helpful in indicating the breakthrough point. The maximum pH value in the effluent (Figure 2c,d) corresponds to a breakthrough, while the minimum pH can be observed at exhaustion due to the increase in metal ions’ concentration and their hydrolysis. The curves for the (Cd + Zn) system (Figure 2d) are much steeper, with a significant decrease in pH values for one pH unit. In the (Pb + Zn) system (Figure 2c), this is not the case and the decrease in pH is not so significant as to provide detection of the breakthrough point by a simple and fast pH measurement. From the breakthrough curves for each metal ion in the (Pb + Zn) system (Figure 3), and for the (Cd + Zn) system (Figure 4), it can be observed that Pb, Cd, and Zn ions bind simultaneously up to breakthrough due to a higher amount of available active binding sites. From Figure 3, it can be seen that Pb does not reach its influent concentration for all examined Pb/Zn ratios, while Zn overshoots the value of *c*/*c*_o_ = 1 in all cycles, which indicates displacement of bound Zn by Pb from the feeding solution. This is the consequence of competition between these two metals, which occurs due to a gradual increase of occupancies of active sites. Namely, when two components are present in a solution, they compete for the available sorption sites, where the component with the higher affinity displaces the one with the lower affinity, causing an overshooting phenomenon [48]. In Figure 4, it can be seen that Cd does not reach its influent concentration except at the highest Cd/Zn ratio, while the Zn concentration in the effluent is again higher than its influent concentration for all Cd/Zn ratios, indicating the displacement effect even in this binary system.

**Figure 5:**
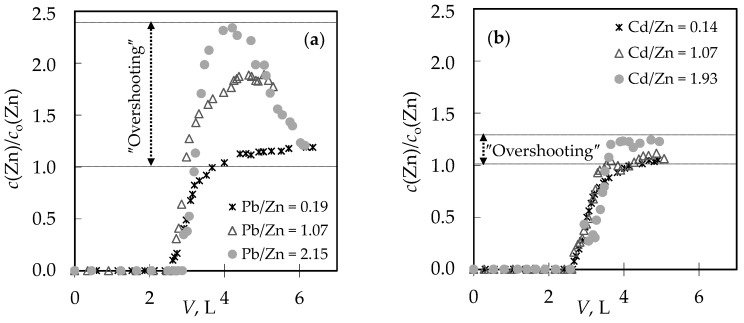
Comparison of Zn breakthrough curves in binary: (**a**) (Pb + Zn) and (**b**) (Cd + Zn) systems at different Pb/Zn and Cd/Zn concentration ratios.

To clarify the behaviour of Zn in the presence of Pb and Cd ions in binary systems, Figure 5 shows the breakthrough curves of Zn ions for different Pb/Zn and Cd/Zn concentration ratios.

In the presence of Pb, Zn exceeded its influent concentration (Figure 5a) by 1.2–2.3 times. This quite significant overshooting can be attributed to the smaller hydrated ionic radius of Pb, and thus the higher affinity. This finding is in agreement with those reported by other researchers [24,44]. The overshooting by 1.1–1.2 times (Figure 5b) in the presence of Cd was quite surprising, especially in the case of the lower initial Cd concentration, because the affinities of those two metal ions are somewhat closer due to a very similar hydrated ionic radius, electronegativity, and energy of hydration [29,49,54,55]. An overshooting in the Zn exit concentration in the presence of Cd was also observed on a *Sargassum algal* bio-sorbent by Figueira et al. 2000, but not on a *Sargassum fluitans* bio-sorbent by Naja et al. 2006 [45,47]. As far as we know, there are no reported results on natural zeolites.

From the breakthrough curves in Figure 2a,b and in Figure 3 and Figure 4, the capacities in breakthrough (*q*_B_) and in exhaustion (*q*_E_) have been calculated using Michael’s method [41] and are presented in Table 1. The column efficiency (*η*) in Table 1, calculated as the ratio of *q*_B_ and *q*_E_, was similar for both systems, indicating that no reduction in sorption efficiency was observed for the six performed sorption-desorption cycles. Ratios of capacities for Pb and Zn in the (Pb + Zn) system and for Cd and Zn in the (Cd + Zn) system in breakthrough overlap with Pb/Zn and Cd/Zn concentration ratios in the feeding solutions, confirming simultaneous binding up to breakthrough. Ratios of capacities in exhaustion are higher than the Pb/Zn and Cd/Zn ratios, indicating better binding of Pb and Cd compared to Zn, as well as the displacement effect. These results are in correspondence with the results obtained for the (Pb + Zn) system at zeolite bed depths of 4 and 12 cm [41].

### 3.2. Analysis of the Regeneration Curves in the Binary (Pb + Zn) and (Cd + Zn) Systems

**Figure 6:**
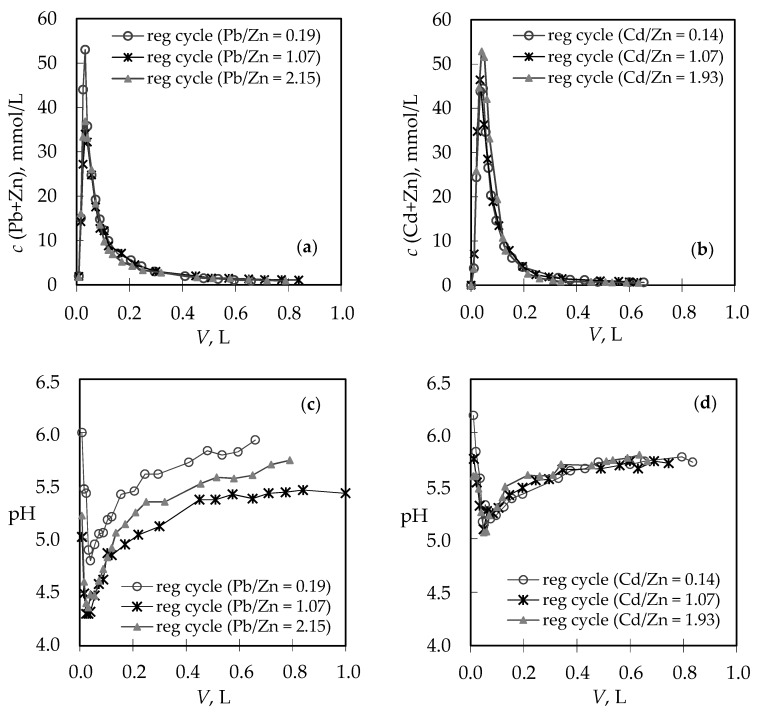
Regeneration curves for different Pb/Zn and Cd/Zn concentration ratios expressed as: (**a**,**b**) Effluent concentrations versus volume; (**c**,**d**) pH changes during regeneration cycles versus volume.

The column’s performance allows for the recovery of the saturated zeolite bed and its reuse through regeneration cycles. Regeneration curves obtained after each service cycle are presented in Figure 6 as effluent concentrations versus volume (Figure 6a,b) and as pH changes during regeneration cycles versus volume (Figure 6c,d).

Regeneration is completed when the (Pb + Zn) and (Cd + Zn) concentrations in the effluent decrease below the initial one in the previous service cycles (Figure 6a,b) and when pH values remain constant (Figure 6c,d). For the (Pb + Zn) binary system, regeneration ended after *t*_R_ ≈ 9–14 h when *V*_R_ ≈ 0.59–0.84 L of the NaNO_3_ solution was spent (Table 2). For the (Cd + Zn) binary system, complete regeneration of the zeolite layer was achieved earlier, after *t*_R_ ≈ 5–8 h with the consumption of a smaller amount *V*_R_ ≈ 0.31–0.49 L of the NaNO_3_ solution (Table 2), confirming higher affinity toward Pb. This can be explained by the fact that Pb, due to the smaller hydrated ionic radius compared to Cd and Zn, can easily access the harder available sites in the zeolite structure. Its desorption is, therefore, more difficult and takes a longer time and a higher volume of the regenerating agent [39,56]. Regeneration was very fast, and resulted in up to ≈5–13 times smaller volumes of effluents compared to the volumes of heavy metal solutions treated in the service cycles. Thus, the recovery of Pb, Cd, and Zn concentrations was very high. This enables their removal from concentrated desorption solutions by some classical treatment process, such as chemical precipitation, or allows for metal reuse; however, this requires a completely different process or a sequence of operations [31,39].

**Figure 7:**
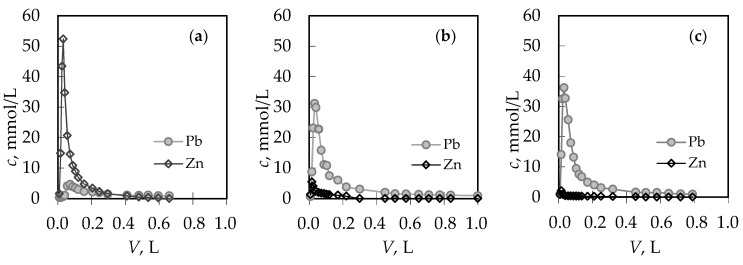
Regeneration curves for each metal ion obtained after service cycles with different Pb/Zn ratios: (**a**) 0.19; (**b**) 1.07; (**c**) 2.15.

**Figure 8:**
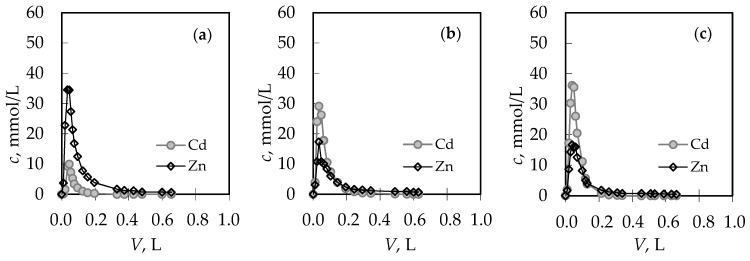
Regeneration curves for each metal ion obtained after service cycles with different Cd/Zn ratios: (**a**) 0.14; (**b**) 1.07; (**c**) 1.93.

The regeneration curves for each metal ion obtained after service cycles have been presented in Figure 7 and Figure 8, while the characteristic parameters of all regeneration curves have been calculated [41] and are summarized in Table 2. The quantity of eluted Pb ions in the (Pb + Zn) system (Figure 7) and Cd ions in the (Cd + Zn) system (Figure 8) increases with the increase in Pb and Cd initial concentration, while the quantity of eluted Zn ions decreases, which was expected. The ratios of *n*_R_(Pb)/*n*_R_(Zn) in Table 2 best confirm the displacement effect since they are significantly higher than the Pb/Zn ratios in the feeding solutions, and more pronounced with the increase of the Pb initial concentration. For the (Cd + Zn) system, those differences are not so significant, which is probably a consequence of the lower overshooting phenomenon explained previously in Figure 5. Regeneration is successfully performed when the recovery ratio (*α_R_*: the ratio of the molar quantity *n*_R_ of ions eluted during regeneration and the molar quantity *n*_E_ of ions bound during the service cycle) is close to 1. Its values (Table 2) higher than 1 confirm successful regeneration but also indicate that the binding of ions continued even after the exhaustion point.

### 3.3. Comparison of the Amount of Ions Removed During Service and Regeneration Cycles

**Figure 9:**
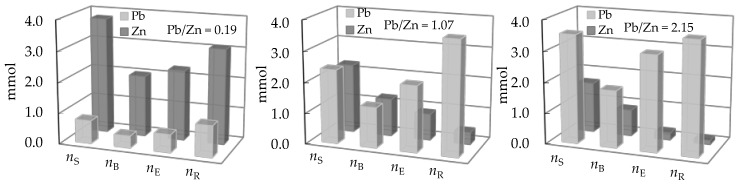
Comparison of *n*_S_, *n*_B_, *n*_E_, and *n*_R_ for different Pb/Zn ratios in the feeding (Pb + Zn) solution.

**Figure 10:**
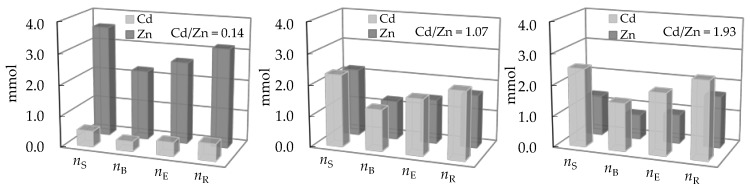
Comparison of *n*_S_, *n*_B_, *n*_E_, and *n*_R_ for different Cd/Zn ratios in the feeding (Cd + Zn) solution.

The comparison of the quantity of ions (*n*_S_) in the feeding solution, bound onto zeolite up to breakthrough (*n*_B_) and exhaustion (*n*_E_), and the quantity of ions eluted (*n*_R_) from the zeolite bed during the regeneration cycle for both binary systems are shown in Figure 9 and Figure 10.

These graphs summarize the obtained results and give the best overview of how the initial concentration of Pb, Cd, and Zn in the feeding (Pb + Zn) and (Cd + Zn) binary solutions can affect *n*_B_, *n*_E_, and *n*_R_ values and consequently the quantity of bound metal ions. For Pb/Zn = 0.19 and Cd/Zn = 0.14, a higher quantity of bound Zn has been obtained, although Zn generally showed lower affinity toward zeolite relative to Pb and Cd. Although clinoptilolite has higher selectivity toward Pb [26,27,29,57,58], this study indicates that the preferential removal of Pb or Cd compared to Zn can be changed by initial concentrations.

### 3.4. Qualitative Evaluation of the Overall Mass Transfer Coefficient in the (Pb + Zn) and (Cd + Zn) Binary Systems

**Figure 11:**
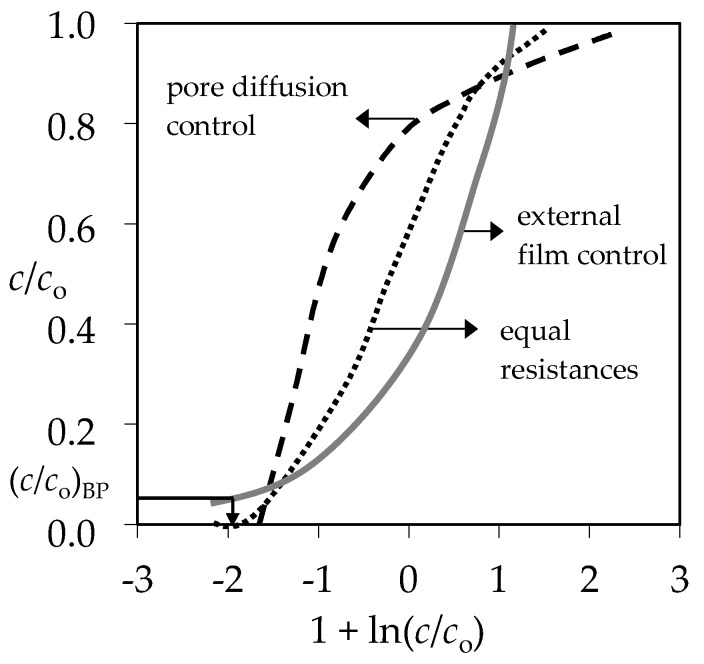
The graphical dependence of effluent and influent solute concentrations ratio versus 1 + ln(*c*/*c*_o_) [60]. Note: (*c*/*c*_o_)_BP_ represents the breakthrough point.

For qualitative evaluation of the controlling mechanism in the fixed bed, the approximate method has been used [59,60,61]. It requires only the experimental column data, specifically the set of effluent concentrations and the corresponding service time. The graphical dependence of *c*/*c*_o_ versus 1 + ln(*c*/*c*_o_) in Figure 11 is suggested.

**Figure 12:**
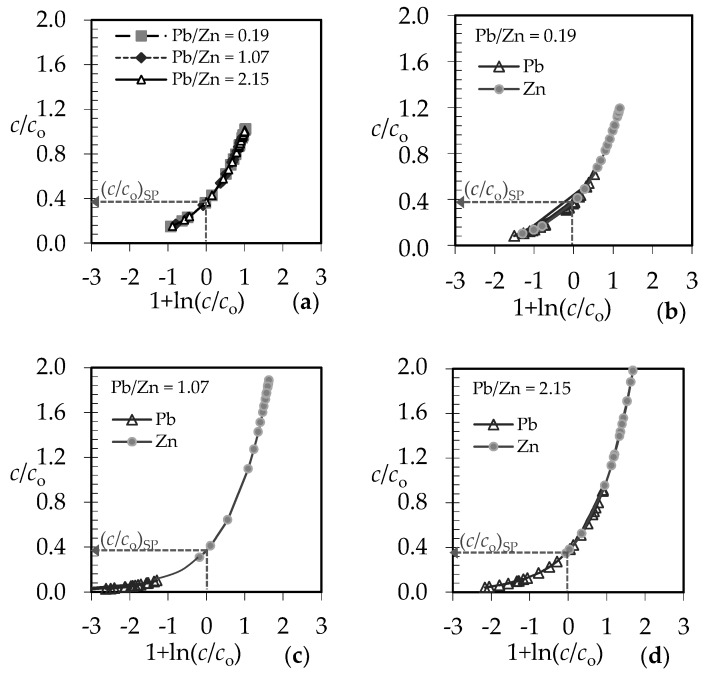
Qualitative evaluation of the rate-controlling step for (Pb+Zn) binary systems at different Pb/Zn ratios: (**a**) for the total (Pb+Zn) concentration; (**b**–**d**) for each ion in the (Pb+Zn) system.

**Figure 13:**
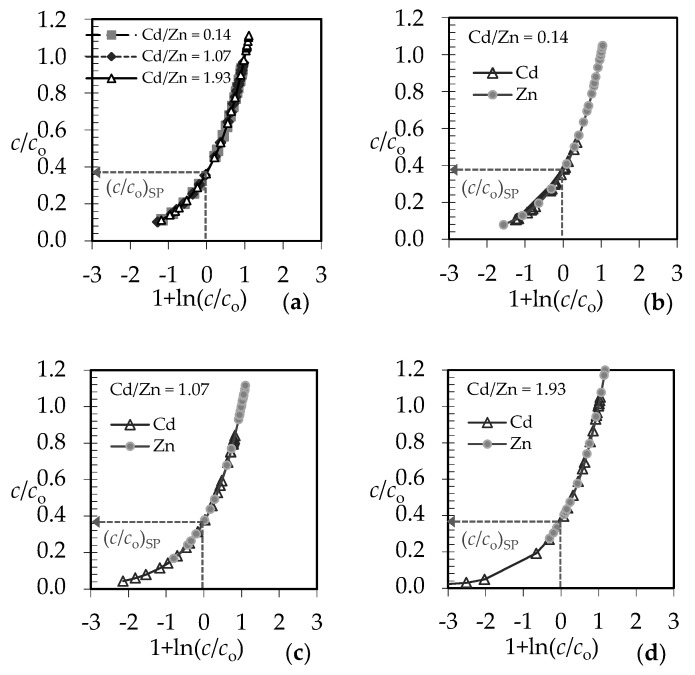
Qualitative evaluation of the rate-controlling step for (Cd + Zn) binary systems at different Cd/Zn ratios: (**a**) for the total (Cd + Zn) concentration; (**b**–**d**) for each ion in the equimolar (Cd + Zn) system.

From the point on the x-axis where 1 + ln(*c*/*c*_o_) equals zero, the value of *c*/*c*_o_ on the y-axis can be determined, and is called the stoichiometric point (*c*/*c*_o_)_SP_. This is the point where the amount of the solute that has passed through the fixed bed equals exactly the residual unfilled capacity of the solid contained before that point. The shape of the breakthrough curve gives the information about the rate-controlling step. The *c*/*c*_o_ values in the stoichiometric point for solid diffusion control systems are in the range of 0.51–0.70, and for liquid film diffusion control systems in the range of 0.31–0.50 [61]. The graphical plots in Figure 12 and Figure 13 represent the ratios of effluent and influent solute concentrations versus 1 + ln(*c*/*c*_o_) for both examined binary systems.

According to the shape of the obtained breakthrough curves, the (*c*/*c*_o_)_SP_ values for both binary systems at different Pb/Zn and Cd/Zn concentration ratios are in the range 0.37–0.39, indicating that the liquid film diffusion is the slowest step and controls the overall process rate [61].

### 3.5. Quantitative Determination of the Overall Mass Transfer Coefficient in the (Pb + Zn) and (Cd + Zn) Binary Systems

The mass transfer coefficient is a function of the physicochemical properties of the pollutant and the medium, the packing material’s properties, and the process conditions. It is a combination of the different partial intrinsic mass transfer coefficients, which is directly related to the effective interfacial area and needs to be evaluated to understand the phenomena of mass transfer in a fixed bed column for heavy metal treatment [62]. The overall mass transfer coefficient *K*_a_ (kg/min m^3^) has been calculated for total (Pb + Zn) and (Cd + Zn), and for each metal ion in the binary systems, using the following equation [40,60,63]:

*K*_a_ = (*N* · *G*_W_)/*H* (1)

where *N* is the overall number of mass transfer units (-), *G*_W_ is the mass flux of the solution in the column (kg/min m^2^), and *H* is the fixed bed of zeolite in the column (m).

It is suggested to determine the value of N from the graphical dependence of *c*/*c*_o_ versus 1 + ln(*c*/*c*_o_) in Figure 12 and Figure 13, where 1 + ln(*c*/*c*_o_) = *N* (*τ* − 1). At the breakthrough point (*c*/*c*_o_)_BP_ in Figure 11, where *c*/*c*_o_ ≈ 0.05, *N* has been calculated from the graphical plots in Figure 12 and Figure 13 according to the equation:

*N* = [1 + ln(*c*/*c*_o_)_BP_]/(*τ* − 1) (2)

where *τ* is the dimensionless time (-) and has been calculated as [33]:

*τ* = *t*_B_/*t*_E_ (3)

The mass flux of the solution in the column has been calculated as follows:

*G*_W_ = (*Q* · *ρ*)/(*A* · ε) (4)

where *Q* is the flow of the solution through the column (m^3^/min), *A* is the cross-sectional area of the column (*A* = 0.00011304 m^2^), *ρ* is the density of water at 25 °C (*ρ* = 997.13 kg/m^3^), and *ε* is the fixed bed porosity (-).

All calculated parameters are listed in Table 3.

The obtained *K*_a_ values in Table 3 and, therefore, the overall mass transfer are higher in the (Cd + Zn) system compared to the (Pb + Zn) one for the same experimental conditions. The reason for this is probably much less pronounced competition between Cd and Zn ions in the (Cd + Zn) binary system compared to Pb and Zn in the (Pb+ Zn) binary system, due to similar physicochemical properties of Cd and Zn ions. The mass transfer of Zn ions decreases with increasing Cd or Pb concentrations in the influent, since this contributes to the increase in ion competition and displacement of bound Zn. Namely, the rate of mass transfer is directly proportional to the area available for transfer and the driving force for the transfer process. Thus, reducing the thickness of the boundary layer or increasing the diffusion coefficient in the film, as a result of the reduced competition and ion migration, enhanced the value of *K*_a_, which consequently improved the rate of the overall mass transfer.

### 3.6. Scanning Electron Microscopy and Energy Dispersive Spectroscopy (SEM-EDS) Results

**Figure 14:**
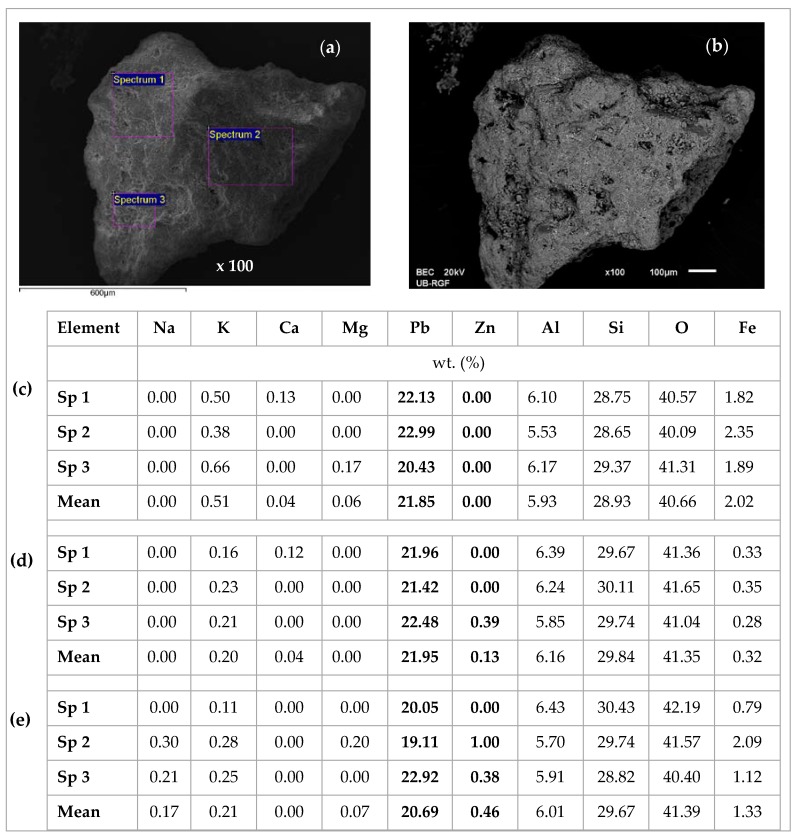
SEM images of the first zeolite grain for the (Pb + Zn) system and Pb/Zn = 1.07: (**a**) SE image of the first grain with three spectrums (Sp) selected for EDS analysis; (**b**) Back-Scattered Electron (BSE) image of the first grain; (**c**) EDS analysis results of the first grain; (**d**) EDS analysis results of the second grain; (**e**) EDS analysis results [42] of the third grain.

**Figure 15:**
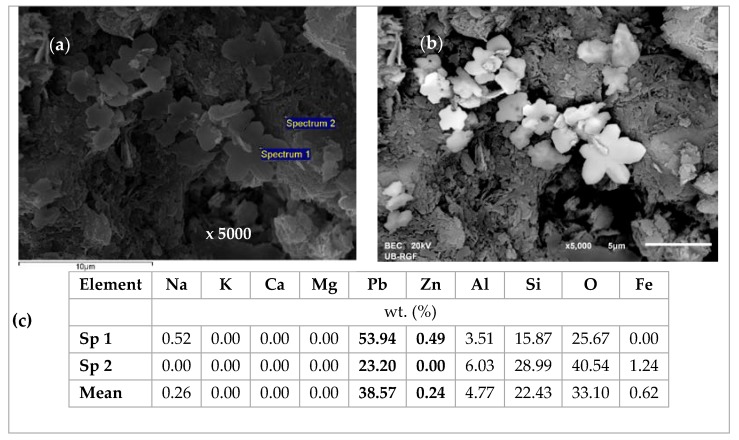
SEM images of white crystals for the (Pb + Zn) system and Pb/Zn = 1.07: (**a**) SE image x 5000 with two spectrums (Sp); (**b**) BSE image x 5000; (**c**) EDS analysis results.

**Figure 16:**
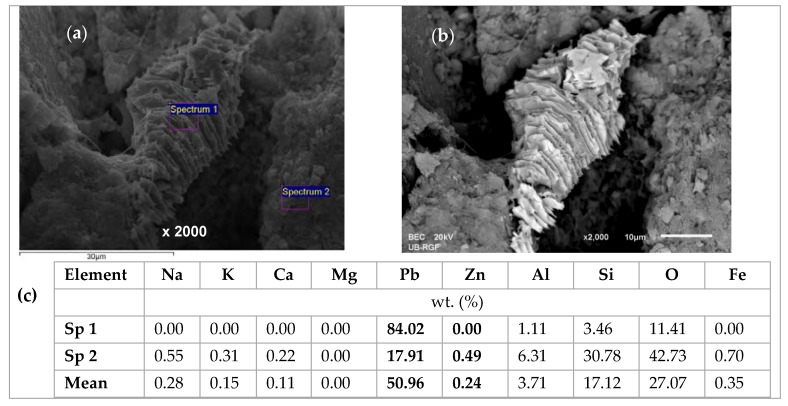
SEM images of crystal for the (Pb + Zn) system and Pb/Zn = 1.07: (**a**) SE image x 2000 with two spectrums (Sp); (**b**) BSE image x 2000; (**c**) EDS analysis results.

**Figure 17:**
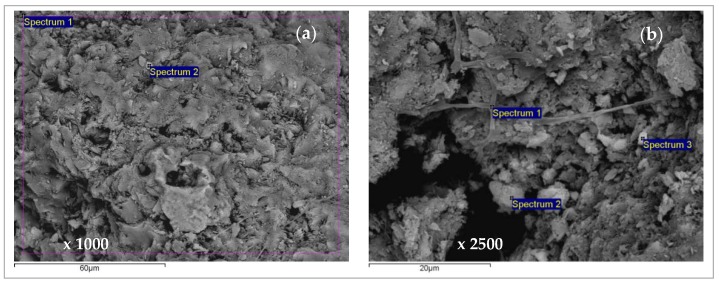
SEM images of zeolite grains for the (Cd + Zn) system and Cd/Zn = 1.07 with: (**a**) two spectrums with mean wt.(%) of 4.62 Cd and 2.22 Zn; (**b**) three spectrums with mean wt.(%) of 3.52 Cd and 2.63 Zn.

**Figure 18:**
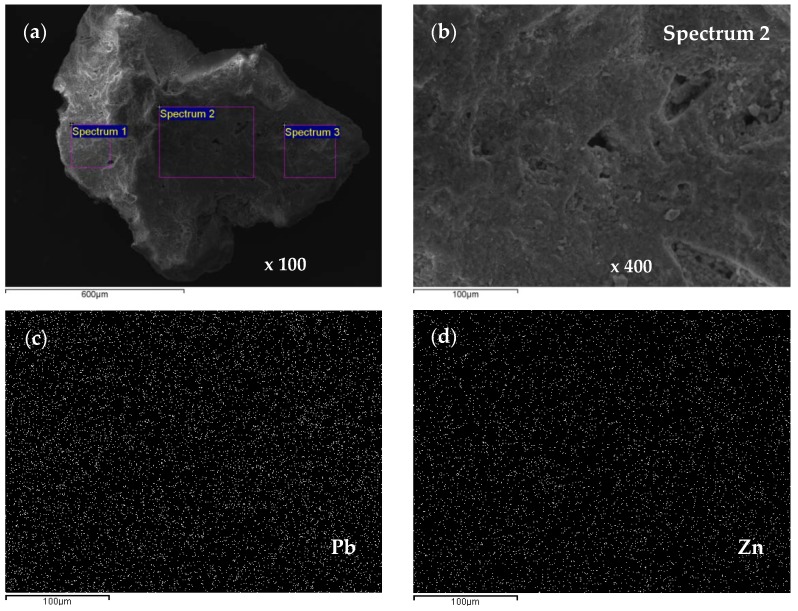
(**a**) An SEM image of a zeolite grain with three spectrums for the (Pb + Zn) system and Pb/Zn = 1.07; (**b**) spectrum 2 with a magnification of 400; (**c**,**d**) a mapping analysis across the spectrum 2 surface with a mean wt.(%) of 20.06 Pb and 0.76 Zn.

**Figure 19:**
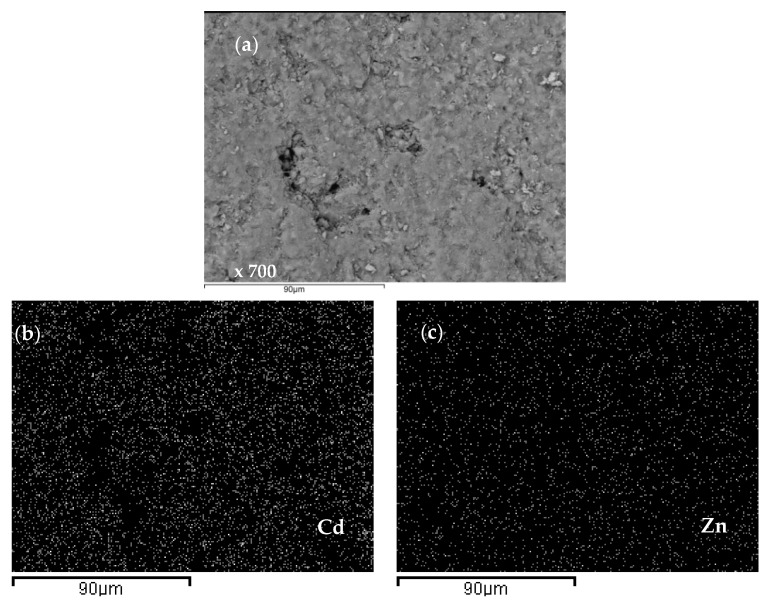
(**a**) An SEM image of the surface on a zeolite grain for the (Cd + Zn) system and Cd/Zn = 1.07, (**b**,**c**) a mapping analysis of the surface with a mean wt.(%) of 5.61 Cd and 3.52 Zn.

Figure 14, Figure 15, Figure 16, Figure 17, Figure 18 and Figure 19 show SEM micrographs and EDS results of zeolite grains taken from the top of the exhausted zeolite layer in the column immediately before regeneration cycles, for both equimolar binary systems (Pb/Zn = Cd/Zn = 1.07). The Secondary Electron (SE) images give an insight into the sample morphology, while in the Back-Scattered Electron (BSE) images the phases of various chemical compositions and the chemical pattern of the sample can be observed. An SEM analysis was used to examine the zeolite particle surface morphology, while semi-quantitative EDS was used to examine the elemental composition of selected areas on the zeolite grains and identify specific elements, especially Pb, Cd, and Zn, as well as their proportions onto zeolite surfaces via a mapping analysis.

From Figure 14 it can be noticed that the EDS analyses of all three grains are very similar, confirming the uniform chemical composition. The lead is bound in a significantly larger amount compared to zinc, although this is an equimolar solution with the same initial lead and zinc concentration. This confirms the higher affinity of lead compared to zinc. The content of exchangeable cations Na, K, Ca, and Mg is much lower than in the raw zeolite sample analyzed in our previous study [53], which can be attributed to the exchange with Pb, Cd, and Zn ions. In addition, the EDS analysis of the same zeolite sample milled to powder obtained a semi-quantitative elemental composition similar to that on the particle surface [42] with the dominant Pb content relative to the other exchangeable cations, confirming that the ion exchange is the main mechanism of ion binding and takes place within the whole zeolite particle [53]. In Figure 14b, rare randomly distributed white agglomerates have been noticed on the zeolite surface and are shown in Figure 15a,b with magnifications of 5000x. The agglomerates are also observed on other zeolite grains, one of which is shown in Figure 16a,b with magnifications of 2000x. The EDS analysis of these agglomerates (in Figure 14c and Figure 15c) found an exceptionally high content of Pb (from ≈54 up to 84 wt. %). This can be explained by higher affinity and thus increased concentrations of Pb at the active sites, when crystallization centres were created and lead hydroxide crystals were formed. It can be assumed that this is due to the complex lead sorption mechanism, including ion exchange, surface complexation, and co-precipitation [64]. The very shape of these agglomerates points to slow crystallization under non-mixing conditions, which applies to the column process. According to the obtained results in these agglomerates, zinc is identified in a very low mass fraction (Figure 15) or is not identified at all (Figure 16, Spectrum 1).

The SEM-EDS analysis for the equimolar (Cd + Zn) system (Figure 16) showed a greater amount of bound Cd compared to Zn in all grains, but not as much as Pb compared to Zn in the (Pb + Zn) system. Also, on the zeolite surface saturated with Cd and Zn, there were no agglomerates as in the case of zeolite saturated with Pb and Zn. Since the EDS analysis of all samples showed a uniform composition and domination of Pb or Cd over Zn, only the content of heavy metals has been highlighted in the following figures.

The distribution of Pb, Cd, and Zn ions onto the zeolite surface in the two equimolar binary systems was also analysed using EDS mapping and the obtained results are presented in Figure 18 and Figure 19.

The obtained results revealed that Pb and Zn in the (Pb + Zn) system as well as Cd and Zn in the (Cd + Zn) system are equally distributed on the zeolite surface, but still with a noticeably greater amount of bound Pb and Cd compared to Zn. These findings are in accordance with the quantity of eluted ions *n*_R_ presented in Figure 9 and Figure 10 for equimolar binary systems.

## 4. Conclusions

Lead, cadmium, and zinc ions have been successfully removed on the fixed zeolite bed from binary (Pb + Zn) and (Cd + Zn) aqueous solutions with different Pb/Zn and Cd/Zn concentration ratios, attaining great efficiencies. This study significantly contributes to understanding the exchange of a particular ion between the breakthrough point and exhaustion, as well as interaction of ions in view of different affinities, and physical and chemical properties. The literature usually compares capacities for different metal ions in the equilibrium stages, without a full analysis of each experiment and a quantification of the mass transfer coefficients in the fixed bed reactor. The results confirm a better sorption capacity for Pb or Cd compared to Zn, but also explain the overshooting phenomenon, which is quite different depending on initial concentrations. This finding is very helpful for the prediction of removal of ions with different properties onto natural zeolites, and also for the treatment of complex polluted aquatic systems in nature. The knowledge of the time when overshooting appears is of great importance for the determination of the point when the feeding solution should be directed to a freshly packed sorbent.

Very similar physical and chemical properties of Cd and Zn resulted in a quite unexpected displacement effect. Despite the reported higher selectivity of clinoptilolite toward Pb or Cd, this research has shown that their preferential removal can be controlled by initial concentrations. It is a very important finding for the future investigations of multi-stage processes. The overall mass transfer coefficients are higher in the (Cd + Zn) system compared to the (Pb + Zn) one for the same experimental conditions, due to less pronounced competition between Cd and Zn and thus smaller resistance.

The SEM-EDS analysis of the zeolite grain surface after saturation for the equimolar (Pb + Zn) and (Cd + Zn) systems confirmed higher affinity of Pb and Cd compared to Zn, even when the initial ions concentrations were almost the same. Moreover, for the (Pb + Zn) system, randomly distributed agglomerates with significant amounts of Pb (up to 84 wt.%) with no identified Zn were found on the zeolite surface. This can be attributed to the complex Pb sorption mechanism, including ion exchange, surface complexation, and co-precipitation, while the very shape of these agglomerates points to slow crystallization under non-mixing conditions, characteristic for the column process. The mapping analysis revealed equal distribution of all three heavy metals across the zeolite surface, but again with the dominant content of bound Pb and Cd compared to Zn.

Successful regeneration of the fixed zeolite bed after saturation enabled its reuse for six consecutive service cycles. Since there was no noticeable loss in sorption capacity, the same zeolite layer can be used further. This research confirms ion exchange as the main mechanism and zeolite as the most promising cost-effective material in the treatment of heavy metal polluted waters, and aims in the selection of natural ion exchangers for multi-component water treatments.

## Figures and Tables

**Table 1:** The parameters of service cycles calculated from breakthrough curves for the (Pb + Zn) and (Cd + Zn) binary systems.

Concentration Ratio in Binary Solutions	*V*_B_ (L)	*t*_B_ (h)	*V*_E_ (L)	*t*_E_ (h)	Metal Ions	*q*_B_ (mmol/g)	*q*_E_ (mmol/g)	*η* (-)	$\frac{q_{B}\;(Pb\;or\;Cd)}{q_{B}(Zn)}$	$\frac{q_{E}\;(Pb\;or\;Cd)}{q_{E}(Zn)}$
Pb/Zn = 0.19	2.62	43.67	4.42	73.58	Pb + Zn	0.404	0.487	0.83	0.19	0.25
					Pb	0.066	0.099			
					Zn	0.338	0.388			
Pb/Zn = 1.07 *	2.72	45.25	4.39	73.08	Pb + Zn	0.430	0.508	0.85	1.07	2.48
					Pb	0.222	0.362			
					Zn	0.208	0.146			
Pb/Zn = 2.15	2.91	48.50	5.12	85.25	Pb + Zn	0.460	0.564	0.82	2.15	13.4
					Pb	0.314	0.525			
					Zn	0.146	0.039			
Cd/Zn = 0.14	2.68	44.58	4.02	67.00	Cd + Zn	0.432	0.519	0.83	0.14	0.17
					Cd	0.053	0.074			
					Zn	0.379	0.445			
Cd/Zn = 1.07	2.67	44.50	4.40	73.33	Cd + Zn	0.437	0.547	0.80	1.07	1.27
					Cd	0.226	0.306			
					Zn	0.211	0.241			
Cd/Zn = 1.93	2.65	44.17	3.91	65.08	Cd + Zn	0.395	0.496	0.79	1.95	2.14
					Cd	0.261	0.338			
					Zn	0.134	0.158			

Note: *V*_B_, *V_E_*: the volumes of the solution treated until breakthrough and exhaustion, respectively; *t_B_*, *t_E_*: the service time in breakthrough and exhaustion, respectively; *q_B_*, *q_E_*: the capacities in breakthrough and exhaustion, respectively; *η*: the column efficiency; * Results from [43].

**Table 2:** The parameters of regeneration cycles for the (Pb + Zn) and (Cd + Zn) binary systems calculated from regeneration curves.

Concentration Ratio in Binary Solutions	*V*_R_ (L)	*t*_R_ (h)	Metal Ions	*n*_R_ (mmol)	$\frac{n_{R}\;(Pb\;or\;Cd)}{n_{R}(Zn)}$	*α*_R_ (-)
Pb/Zn = 0.19	0.59	9.92	Pb + Zn	4.101		
			Pb	1.016	0.33	1.43
			Zn	3.085		
Pb/Zn = 1.07	0.84	14.00	Pb + Zn	4.066		
			Pb	3.675	9.39	1.36
			Zn	0.391		
Pb/Zn = 2.15	0.72	12.00	Pb + Zn	3.791		
			Pb	3.661	28.16	1.14
			Zn	0.130		
Cd/Zn = 0.14	0.43	7.17	Cd + Zn	3.708		
			Cd	0.540	0.17	1.21
			Zn	3.168		
Cd/Zn = 1.07	0.49	8.17	Cd + Zn	3.880		
			Cd	2.182	1.29	1.20
			Zn	1.698		
Cd/Zn = 1.93	0.31	5.17	Cd + Zn	4.163		
			Cd	2.503	1.51	1.42
			Zn	1.660		

*V_R_*: the volume of effluent up to the end of the regeneration cycle; *t_R_*: the time when the regeneration cycle ends; *n*_R_*:* the quantity of ions eluted from the zeolite bed during regeneration; *α_R_*: the recovery ratio.

**Table 3:** The mass transfer parameters for the (Pb + Zn) and (Cd + Zn) binary systems.

Concentration Ratio in Binary Solutions	Metal Ions	τ -	N (-)	*G*_W_ (kg/min m^2^)	*K*_a_ (kg/min m^3^)
Pb/Zn = 0.19	Pb + Zn	0.59	2.27	12.73	361
	Pb		/*		/*
	Zn		3.18		506
Pb/Zn = 1.07	Pb + Zn	0.62	2.08	12.73	304
	Pb		6.30		918
	Zn		0.46		68
Pb/Zn = 2.15	Pb + Zn	0.57	2.05	12.73	326
	Pb		4.15		660
	Zn		0.14		22
Cd/Zn = 0.14	Cd + Zn	0.67	3.48	12.73	554
	Cd		/*		/*
	Zn		4.66		741
Cd/Zn = 1.07	Cd + Zn	0.61	3.26	12.73	518
	Cd		5.45		868
	Zn		2.04		324
Cd/Zn = 1.93	Cd + Zn	0.68	3.68	12.73	586
	Cd		7.82		1244
	Zn		0.34		55

* Too low Cd or Pb concentrations in the breakthrough due to which it was not possible to calculate *N* and *K*_a_.
